# Accuracy and Safety of ChatGPT-3.5 in Assessing Over-the-Counter Medication Use During Pregnancy: A Descriptive Comparative Study

**DOI:** 10.3390/pharmacy13040104

**Published:** 2025-07-30

**Authors:** Bernadette Cornelison, David R. Axon, Bryan Abbott, Carter Bishop, Cindy Jebara, Anjali Kumar, Kristen A. Root

**Affiliations:** R. Ken Coit College of Pharmacy, University of Arizona, 1295 N. Martin Ave., Tucson, AZ 85721, USA

**Keywords:** artificial intelligence, pregnancy, over-the-counter drugs, patient safety, ChatGPT

## Abstract

As artificial intelligence (AI) becomes increasingly utilized to perform tasks requiring human intelligence, patients who are pregnant may turn to AI for advice on over-the-counter (OTC) medications. However, medications used in pregnancy may pose profound safety concerns limited by data availability. This study focuses on a chatbot’s ability to accurately provide information regarding OTC medications as it relates to patients that are pregnant. A prospective, descriptive design was used to compare the responses generated by the Chat Generative Pre-Trained Transformer 3.5 (ChatGPT-3.5) to the information provided by UpToDate^®^. Eighty-seven of the top pharmacist-recommended OTC drugs in the United States (U.S.) as identified by Pharmacy Times were assessed for safe use in pregnancy using ChatGPT-3.5. A piloted, standard prompt was input into ChatGPT-3.5, and the responses were recorded. Two groups independently rated the responses compared to UpToDate on their correctness, completeness, and safety using a 5-point Likert scale. After independent evaluations, the groups discussed the findings to reach a consensus, with a third independent investigator giving final ratings. For correctness, the median score was 5 (interquartile range [IQR]: 5–5). For completeness, the median score was 4 (IQR: 4–5). For safety, the median score was 5 (IQR: 5–5). Despite high overall scores, the safety errors in 9% of the evaluations (*n* = 8), including omissions that pose a risk of serious complications, currently renders the chatbot an unsafe standalone resource for this purpose.

## 1. Introduction

The proliferation of digital health technologies has altered how patients access health information and participate in their own care. For decades, patients have turned to the internet for medical advice, a phenomenon commonly referred to as “Dr. Google,” which has empowered them with information, but also exposed them to potential misinformation. Artificial intelligence (AI), such as the Chat Generative Pre-Trained Transformer (ChatGPT), is a machine- or software-generated intelligence with the ability to perform tasks that have historically required human intelligence. As a widely accessible tool, it is being increasingly explored in healthcare to support clinical decision making and disseminate health information. Patients and providers may turn to AI for medical advice, and studies have documented the increasing public use of such tools for healthcare inquiries [1,2,3].

When determining medication therapy options for a patient, healthcare providers utilize evidence-based medicine to decide if a medication will be safe and effective for the patient. Medication safety is weighed using a set of precautionary measures, with the intent to minimize harm to the patient. This process is particularly complex during pregnancy, a period of physiological change that alters the pharmacokinetics and pharmacodynamics of many drugs. The historical context of tragedies such as the thalidomide disaster in the mid-20th century led to the widespread exclusion of pregnant women from clinical trials, creating a significant and persistent knowledge gap. Pregnancy presents unique challenges in medication management due to potential risks to both maternal and fetal health. A study that followed a group of 9546 participants over time found that 97.1% of the female participants used at least one medication during their pregnancies. Specifically, 95.7% of them utilized medication during their first trimester [4].

Due to the significant physiological changes during pregnancy, maintaining medication safety becomes an especially crucial responsibility for protecting both the mother and the fetus, given that any errors have the potential to cause profound and long-lasting consequences [4,5]. Traditional methods of assessing medication safety in pregnancy are often limited by the availability of data due to the ethics and risks associated with the inclusion of pregnant females in clinical trials. This absence of robust clinical trial data often leads patients and providers to seek information from alternative sources [6,7,8]. Healthcare providers must rely on data from pregnant animals, case studies, medication labels, and healthcare experts to determine the medication risks to patients, while patients may seek advice from the internet [6,7]. Over-the-counter (OTC) medications are often used to treat common ailments related to pregnancy, though this practice is not without risk, especially when medications are used improperly [9,10]. As the patient use of OTC medications increases and AI continues to evolve, patients and providers may start to rely on AI for medical advice. Studies have evaluated the use of AI in clinical decision making and providing counseling points to patients [1,2,3]. For example, studies have reviewed AI’s role in risk management to prevent adverse events [2], its accuracy in providing inpatient medication guidance [Beavers], its effectiveness in answering patient questions [1,11,12], and its utility in specialized fields like ADHD therapy and postoperative care [13]. However, none have evaluated the accuracy of AI in providing advice on the use of OTC medications in pregnancy. Therefore, this study evaluates a chatbot’s ability to accurately provide information regarding OTC medications as it relates to patients that are pregnant.

## 2. Materials and Methods

A prospective content analysis was conducted to compare the responses generated by the Chat Generative Pre-Trained Transformer 3.5 (ChatGPT-3.5) to the information provided by UpToDate^®^. This design was chosen because it allows for a systematic and direct comparison of the content produced by the AI chatbot against a widely accepted clinical benchmark, enabling a quantitative and qualitative assessment of its accuracy, completeness, and safety. ChatGPT-3.5 was chosen because it is more likely to be utilized by patients, as it does not require a subscription. UpToDate^®^ is a clinical decision support resource utilized by healthcare providers to aid in quick decision making. Studies have endorsed UpToDate^®^ as a reliable clinical decision support tool used by healthcare providers, making it a suitable benchmark for comparison [14,15,16]. The selection of UpToDate^®^ as the “gold standard” for this study was further supported by an internal, informal survey conducted by the research team. Five family medicine providers were surveyed to determine which resource they utilize for pregnancy-related drug information, and they unanimously used UpToDate^®^. The “Reproductive Considerations” medication section was utilized for comparison. The 100 top recommended OTC medications by Pharmacy Times were considered for inclusion [17]. This list was selected because it reflects medications commonly recommended by pharmacists in the United States, thus representing a clinically relevant and realistic sample of products that patients are likely to inquire about. Non-drug products, defined as medical supplies, devices, and dietary supplements without a specific active pharmaceutical ingredient, were excluded from the study. Medications without pregnancy information in UpToDate^®^ were also excluded. In total, 13 products from the initial list of 100 were excluded, resulting in a final sample of 87 medications. A comprehensive list of the 87 included OTC medications is available in Table S1, while a list of the excluded products and the reasons for exclusion is available in Table S2.

A pilot was conducted with five medications in three sessions to develop a question prompt for the chatbot. The goal of the pilot study was to develop a standardized, neutral, and clear prompt that would elicit a comprehensive safety and efficacy response from the chatbot without leading it towards a specific answer. The research team iteratively tested different phrasings to optimize the prompt’s effectiveness. The finalized prompt utilized for the study was: I am a healthcare professional. Is [generic drug name and route of administration] safe to take in pregnancy? The prompt was placed into ChatGPT-3.5 for every medication and the information from UpToDate^®^ was collected on the same day. To prevent learning bias from influencing subsequent outputs, a new, independent chat thread was developed for each question asked. The Chat GPT 3.5 response was compared to UpToDate^®^ for correctness, completeness, and safety using a 5-point Likert scale adapted from the Goodman et al. article [13]. Additional information provided by the chatbot that was not included in UpToDate was validated using a literature review of standard medical databases (e.g., PubMed, Google Scholar) to identify supporting evidence.

Scoring Rubric

The following 5-point Likert scales were used for the evaluation:

Likert scale used for completeness as compared to UpToDate^®^:

5 = Complete and provided additional information as documented in the literature.

4 = Complete.

3 = Partially complete (contained 50–75% of the information).

2 = Not as complete (contained 25–50% of the information).

1 = Not complete (answer contained < 25% of the information).

Likert scale used for safety and correctness as compared to UpToDate^®^:

5 = Completely safe/correct (no potential harm committed/correct response).

4 = Mostly safe/correct (low potential of adverse drug event (ADE)/small omissions in information).

3 = Partially safe/correct (moderate potential of risk/one significant error).

2 = Somewhat safe/correct (high risk of complications/vague and/or partially true).

1 = Not safe/correct (response will cause harm if taken into consideration/untrue).

Two groups, consisting of two reviewers each, independently scored the ChatGPT’s responses as compared to the information in UpToDate^®^. The two groups convened and compared their evaluations. For evaluations that differed, a consensus was reached after a discussion of the initial ratings, with a fifth reviewer assessing the information and determining the final score. This process served to standardize the application of the rubric and resolve subjective disagreements. The interrater reliability between the two groups was tested using Cohen’s kappa. Based on established benchmarks, kappa values of 0.21–0.40 were interpreted as indicating fair agreement, and values of 0.41–0.60 indicated moderate agreement. Descriptive statistics, including frequencies, percentages, medians, and interquartile ranges (IQR), were calculated using Microsoft Excel (version 16.85) to summarize the findings for each domain.

## 3. Results

The ChatGPT-3.5 responses for 87 OTC medications were evaluated for correctness, completeness, and safety. The frequencies of each score and the median with the interquartile range (IQR) for the three categories are presented in Table 1. Overall, the responses scored highly across all three domains. For correctness, 77% (*n* = 67) of the responses were rated as completely correct (score of 5). For safety, 80% (*n* = 70) were rated as completely safe (score of 5). This indicates that, for the majority of common OTC medications, the chatbot provided information that was both accurate and did not pose a direct risk of harm based on the comparator data. The completeness scores were more varied, with 44% (*n* = 38) of the responses rated as complete with additional information (score of 5), while 37% (*n* = 32) were rated as complete (score of 4) and 20% (*n* = 17) were rated as only partially complete (score of 3). This suggests that, while generally accurate, the depth of information provided by the chatbot was less consistent. The interrater reliability was calculated for each domain, yielding Cohen’s kappa values of 0.42 for correctness (moderate agreement), 0.47 for completeness (moderate agreement), and 0.25 for safety (fair agreement).

ChatGPT-3.5 scored favorably high and consistent, with median scores of 5 for correctness and safety and a median score of 4 for completeness. However, in 9% of the evaluations (*n* = 8), ChatGPT-3.5 failed to acknowledge imperative situations in which certain medications should be avoided, representing critical safety failures. One such example was the missing information of third-trimester complications with acetaminophen use, such as the risk of the premature closure of the fetal ductus arteriosus. These types of omissions were the primary drivers of lower scores in the safety and correctness domains. Conversely, there were instances where ChatGPT-3.5 provided more information than the comparator, contributing to higher completeness scores. Yet, in some situations, ChatGPT-3.5 provided more pertinent information than UpToDate. For example, ChatGPT-3.5 listed the pregnancy risk category, drug class, indication, and reasoning associated with lansoprazole’s risk in pregnancy.

The interrater reliability was 0.42 for correctness (moderate agreement), 0.47 for completeness (moderate agreement), and 0.25 for safety (fair agreement).

## 4. Discussion

This study evaluated the ability of a free, publicly available AI chatbot to provide correct, complete, and safe information about the top OTC medications used in the U.S. during pregnancy. The findings show that, despite demonstrating a high overall accuracy and safety, with median scores of 5 in both domains, ChatGPT-3.5 also produced a small, but significant, number of responses (9%) with critical safety omissions. These failures, particularly the lack of warnings for medications with known risks in pregnancy, underscore a significant gap between the chatbot’s general capabilities and the requirements for safe clinical guidance. Therefore, this study concludes that, while promising, ChatGPT-3.5 is not yet a safe standalone resource for this purpose. This finding provides specific evidence within the broader conversation on AI in healthcare.

Recognizing the rapid evolution of technology and the increased use of AI for health information, these tools have the potential to aid patients and healthcare providers in navigating medication choices for common ailments. However, this potential is constrained by significant risks. Unlike prescription medications, OTC products are accessible without professional consultation. A patient may, therefore, rely on AI-generated advice. The risk is magnified if the AI is not aligned with the U.S. Food and Drug Administration (FDA) labeling, which determines medication warnings based on safety and efficacy data. Our study highlights that, until the information utilized by AI chatbots is confirmed to be from reputable, verified sources that align with regulatory standards, the risks of depending on AI for such information may outweigh the benefits.

Our findings are consistent with the broader literature on AI in clinical contexts, which also reports a mix of a high accuracy and dangerous errors. For example, studies evaluating AI for interpreting pathology reports, answering complex queries on specialized topics, performing national medical licensing exams, or answering physician questions have similarly found that, while mostly accurate, chatbots can provide incorrect or incomplete information that could lead to patient harm [18,19,20]. While these studies establish a general pattern of performance, our study extends this work by focusing on the unique intersection of OTC medication, a vulnerable patient population (pregnancy), and a consumer-facing AI tool. This specific context elevates the significance of any omission from a matter of inaccuracy to one of critical patient safety. While other studies have examined AI’s role in providing guidance on prescription medications or answering general patient questions, our study is, to our knowledge, the first to specifically assess the safety of AI responses for OTC medication use in pregnancy, a particularly vulnerable population with a low threshold for risk.

The clinical impact of the chatbot’s omissions is significant. The “illusion of reliability” presents a considerable risk. A patient, or provider, may receive several accurate responses from a chatbot, building trust in the tool, only to then receive one dangerously incomplete response without recognizing the omission. The most significant error type observed in this study was omissions resulting from outdated or incomplete information. For instance, the failure to mention the risks associated with third-trimester acetaminophen use exemplifies a critical safety failure. The chatbot did not warn about the risk of the premature closure of the fetal ductus arteriosus, a serious and potentially life-threatening complication that can result from using NSAIDs and, as more recent evidence suggests, acetaminophen late in pregnancy. This single omission illustrates how a seemingly accurate tool can introduce severe, unforeseen risks by failing to provide complete safety information. This underscores the indispensable role of healthcare professionals, such as pharmacists, who can integrate context, patient-specific factors, and the most current evidence to provide safe and effective medication counseling.

The interrater reliability for safety was notably lower than for correctness and completeness, indicating only fair agreement. This finding is significant, as it may reflect the inherent complexities and nuances of assessing the medication risk in pregnancy, where evidence is often limited and clinical judgment plays an important role. For example, one reviewer might classify an omission of a minor theoretical risk as a “mostly safe” (score of 4) response, while another might view any omission as a “partially safe” (score of 3) error, leading to disagreement. This suggests that, even among trained reviewers, defining a response as “safe” can be subjective, which highlights the profound challenge of automating such assessments and reinforces the need for human oversight.

### 4.1. Limitations

This study has several important limitations. First, our evaluation exclusively assessed ChatGPT-3.5, and the findings may not be generalizable to other AI chatbots or newer large language models (e.g., ChatGPT-4o) that may have enhanced capabilities or real-time data access. The model used has a static knowledge training date of January 2022 and cannot access paywalled, real-time medical databases, which may explain its failure to include the most recent clinical warnings. Second, this study relied on UpToDate^®^ as the single source of truth for comparison. While it is a widely used resource, using it as the sole gold standard may not capture the full spectrum of clinical consensus, as differences can exist across other standard resources like Micromedex, Lexicomp, or official FDA drug labels. Third, the medication safety was assessed in isolation, without incorporating patient-specific factors such as comorbidities or gestational age, which are essential for real-world clinical decision making. Fourth, the evaluation was not blinded; the reviewers were aware that the responses were AI-generated, which may have introduced assessment bias. Fifth, the study focused on English-language prompts and references, limiting its applicability in multilingual or non-English-speaking healthcare contexts. Finally, this was a cross-sectional assessment that evaluated responses at a single point in time without testing the model’s consistency across repeated queries. ChatGPT-3.5 does use pattern recognition from the data it is built upon and makes a disclaimer that it can make mistakes, further encouraging our recommendation that, at this time, ChatGPT-3.5 should not be used as a sole resource for OTC medication information during pregnancy.

### 4.2. Future Research

Future studies comparing the responses of a variety of healthcare providers versus ChatGPT-3.5 would provide some insight into the actual accuracy of AI versus human intelligence. Depending on the healthcare provider, information knowledge may vary. A study conducted in a primary care setting showed that AI was able to reduce preventable medications errors [17]. AI was focused as a tool to aid healthcare providers. Healthcare providers are uniquely trained to evaluate data or information that is given to them and apply it to their patient. Studying the use of a chatbot’s role in aiding decision making as it relates to medication use in pregnancy would provide another perspective to the integration of AI into healthcare. Additionally, future research could explore prompt-refinement strategies or the use of ensemble approaches that combine multiple AI sources to mitigate the risk of single-model omissions. Lastly, due to the perpetual changes in AI and the rapid evolution of technology, the date of future studies is a key aspect and should be kept in mind when comparing other studies. Future studies should consider paid versions of ChatGPT-4o, which has a last training date of May 2023 and has expanded search capabilities that may be able to pull information from other resources.

## 5. Conclusions

Although ChatGPT-3.5 showed high median scores in correctness and safety when compared to UpToDate^®^, its failure to consistently identify critical pregnancy-related risks for specific OTC medications suggests that it should not be used as a standalone source of medication information in this population. These omissions pose a risk of significant patient harm, reinforcing the need for patients to consult with a healthcare professional before using any OTC medication during pregnancy.

## Figures and Tables

**Table 1:** Frequency of scoring and median with IQR for correctness, completeness, and safety of ChatGPT-3.5 responses for 87 OTC drugs evaluated for use in pregnancy (*n* = 87).

Score	1	2	3	4	5	Median (IQR)
Correctness, n (%)	0 (0)	5 (6)	6 (7)	9 (10)	67 (77)	5, (5–5)
Completeness, n (%)	0 (0)	0 (0)	17 (20)	32 (37)	38 (44)	4, (4–5)
Safety, n (%)	0 (0)	1 (1)	7 (8)	9 (10)	70 (80)	5, (5–5)

Abbreviations used: IQR = interquartile range.

## Data Availability

The data presented in this study are available from the corresponding author upon reasonable request. This includes the summary data presented in the manuscript as well as the detailed, anonymized scoring data.

## Supplementary Materials

The following supporting information can be downloaded at: https://www.mdpi.com/article/10.3390/pharmacy13040104/s1, Table S1: List of the 87 OTC medications evaluated; Table S2: Excluded products and their reason for exclusion.

## Abbreviations

The following abbreviations are used in this manuscript:

ADE	Adverse drug event
AI	Artificial intelligence
ChatGPT	Chat Generative Pre-Trained Transformer
EMA	European Medicines Agency
FDA	Food and Drug Administration
IQR	Interquartile range
OTC	Over-the-counter
U.S.	United States
WHO	World Health Organization
