# Supplementary material for: Accuracy and Safety of ChatGPT-3.5 in Assessing Over-the-Counter Medication Use During Pregnancy: A Descriptive Comparative Study
Source: Pharmacy (Basel). 2025 Jul 30;13(4):104. doi: 10.3390/pharmacy13040104 (PMC12389367; doi:10.3390/pharmacy13040104)
Supplement: Supplementary file 1 [file pharmacy-13-00104-s001.zip › pharmacy-3727325-supplementary.pdf]

Table S1: List of the 87 OTC medications evaluated

acetaminophen
Acetaminophen, aspirin, caffeine
Acetaminophen, Caffeine, Pyrilamine Maleate
Acetaminophen, Chlorpheniramine, Phenylephrine
Acetaminophen, dextromethrophan, phenylephrine
Acetaminophen, diphenhydramine
Acetaminophen, guai[illegible]enesin, phenylephrine
Acetaminophen, Pamabrom, Pyrilamine maleate
Activated Charcoal
adapalene
Aluminum hydroxide, magnesium carbonate
Aluminum hydroxide, magnesium hydroxide, simethicone
aspirin
azelastine
Benzocaine, resorcinol
Benzoyl peroxide
Benzyl Alcohol, Camphor, Menthol
bisacodyl
bismuth subsalicylate
Calamine, Benzoyl Alcohol, Diphenhydramine
calcium carbonate

Calcium Polycarbophil
Camphor, Eucalyptus, Menthol
Capsaicin
cetirizine, pseudoephedrine
cetirzine
Chlorpheniramine, Dextromethorphan
cimetidine
clotrimizaole
Cromolyn Sodium
DEET
dextromethorphan
diclofenac
Dimenhydrinate
Dimethicone
diphenhydramine
docusate
doxylamine
Doxylamine, acetaminophen, dextromethorphan
Doxylamine, Dextromethorphan
esomeprazole
famotidine
fexofenadine

Fexofendadine, pseudoephedrine
fluticasone
guaifenacin & dextromethorphan
Guaifenacin, pseudoephedrine
guaifenesin
hydrocortisone
ibuprofen
Ibuprofen, diphenhydramine
Ibuprofen, pseudoephedrine
Ketoconazole
ketotifen fumarate
lansoprazole
levocetirizine
Levonorgestrel
Lidocaine
loperamide
loratadine
loratadine, pseudoephedrine
Meclizine
Methyl salicylate
miconazole
naproxen

Naproxen, diphenhydramine
nicotine gum
nicotine lozanges
nicotine patches
olopatadine
Omeprazole
Oxymetazoline
PEG 3350
permethrin
phenazopyridine
phenylephrine
piperonyl butoxide, pyrethrum extract
pseudoephedrine
psyllium husk
Salicylic acid
Selenium Sulfide
senna
Senna and docusate
simethicone
terbinafine
triamcinolone
Wheat Dextrin

Table S2: Excluded products and their reason for exclusion.

**Medications**	**Reason for Exclusion**
alpha galactosidase enzyme	Lacking pregnancy information in UpToDate®
Alpha-lipoic acid, B-vitamins	Non-drug product
Calamine, Pramoxine	Lacking pregnancy information in UpToDate®
carbamide peroxide	Lacking pregnancy information in UpToDate®
Carboxymethylcellulose	Lacking pregnancy information in UpToDate®
docosanol	Lacking pregnancy information in UpToDate®
lactase enzyme	Lacking pregnancy information in UpToDate®
melatonin	Non-drug product
Menthol and Camphor	Non-drug product
Naphazoline Hcl, Pheniramine maleate	Lacking pregnancy information in UpToDate®
Pyrithione Zinc	Lacking pregnancy information in UpToDate®
Tetrahydrozoline	Lacking pregnancy information in UpToDate®
Tolnaftate	Lacking pregnancy information in UpToDate®
